# Hospital admissions following emergency medical services in Germany: analysis of 2 million hospital cases in 2022

**DOI:** 10.1007/s00063-024-01148-6

**Published:** 2024-04-23

**Authors:** Martin Roessler, Claudia Schulte, Christoph Bobeth, Danny Wende, Christian Karagiannidis

**Affiliations:** 1 BARMER Institute for Health Care System Research, Axel-Springer-Str. 44, 10969 Berlin, Germany; 2 ARDS and ECMO Centre Cologne-Merheim, Cologne, Germany; 3 University Witten/Herdecke, Witten, Germany

**Keywords:** Emergency medical services, Helicopter emergency medical services (HEMS), Emergency department, Resuscitation, Costs, Rettungsdienst, Medizinische Notfalldienste per Hubschrauber, Notaufnahme, Wiederbelebung, Kosten

## Abstract

**Background:**

The use of emergency medical services (EMS) in Germany has increased substantially over the last few decades. While current reform efforts aim to increase effectiveness and efficiency of the German hospital and EMS systems, there is lack of data on characteristics of hospital cases using EMS.

**Objectives:**

To analyze and compare the characteristics of cases hospitalized with and without the use of EMS.

**Materials and methods:**

The BARMER health insurance data on more than 2 million hospital cases admitted in 2022 were analyzed. The distributions of age, clinical complexity (measured by patient clinical complexity levels, PCCL), main diagnoses, costs for EMS and hospital treatment, and multiple severity indicators were described. The overall severity of hospital cases was classified as “low or moderate” or “high” based on a combined severity indicator. All analyses were stratified by use of EMS and EMS type.

**Results:**

A total of 28% of all included hospital cases used EMS. Relative to hospital cases without use of EMS, hospital cases with use of EMS were older (physician-staffed ambulance: 75 years, interquartile range [IQR] 59–84, double-crewed ambulance: 78 years, IQR 64–85) and had a higher clinical complexity. The severity of more than 30% of the cases using EMS (except for patient transport service ambulance) was classified as “low or moderate”. The distributions of main diagnoses differed by severity and use of EMS.

**Conclusions:**

The high proportion of cases with low or moderate severity using EMS may indicate a substantial potential to avoid the use of EMS in the context of hospital admissions in Germany. Further investigation is required to explore whether the proportion of cases using EMS could be reduced by optimizing preclinical service.

## Abstract (de)

**Hintergrund:**

In den vergangenen Jahrzehnten war eine deutliche Steigerung der Inanspruchnahme des Rettungsdiensts (RD) in Deutschland zu verzeichnen. Während aktuelle Reformbestrebungen auf eine Erhöhung der Effektivität und Effizienz der Krankenhausversorgung und des Rettungsdiensts zielen, fehlen Daten zu Charakteristika von Krankenausfällen mit Inanspruchnahme des RD.

**Ziel der Arbeit:**

Analyse und Vergleich der Charakteristika von Krankenhausfällen mit und ohne Inanspruchnahme des RD.

**Material und Methoden:**

Die BARMER-Daten zu mehr als 2 Mio. Krankenhausfällen im Jahr 2022 wurden hinsichtlich Alter, „patient clinical complexity level“ (PCCL), Hauptdiagnosen, Kosten sowie Schweregradindikatoren analysiert. Der Schweregrad eines Falls wurde auf Grundlage eines kombinierten Schweregradindikators als „niedrig bis moderat“ oder „hoch“ eingestuft.

**Ergebnisse:**

Insgesamt 28 % aller Krankenhausfälle ging eine Inanspruchnahme des RD voraus. Im Vergleich zu Krankenhausfällen ohne Einsatz von Rettungsdiensten waren Krankenhausfälle mit Einsatz von Rettungsdiensten älter (Notarztwagen: 75 Jahre, Interquartilsabstand [IQR] 59; 84; Rettungswagen: 78 Jahre, IQR 64; 85) und hatten eine höhere klinische Komplexität. Der Schweregrad von mehr als 30 % der Fälle mit RD-Nutzung (exklusive Krankentransportwagen) wurde als „niedrig bis moderat“ eingestuft. Die Hauptdiagnosen unterschieden sich nach Schweregrad und Inanspruchnahme des RD.

**Diskussion:**

Der hohe Anteil an Fällen mit niedrigem bis moderatem Schweregrad, die den RD in Anspruch nahmen, könnte auf ein relevantes Potenzial zur Vermeidung der RD-Nutzung im Kontext von Krankenhausaufnahmen in Deutschland hindeuten. Weitere Untersuchungen sind notwendig, um zu prüfen, ob der Anteil dieser Fälle mit Nutzung des RD durch eine Optimierung der präklinischen Versorgung reduziert werden kann.

## Background

The use of prehospital emergency medical services (EMS) in Germany has increased dramatically over the past few decades [5]. Increasing numbers of hospitalizations and higher costs associated with the increased use of EMS implies a rising burden for hospitals, ambulance services, and the German health care system in general.

The German government therefore sat up a commission in 2021 to draw up reform proposals for hospital reform and the reform of emergency care. The proposals are the subject of current health policy discussions. However, despite that there is substantial need for reform of the German hospital and EMS systems, there is a lack of data on characteristics of cases using EMS in the context of hospital admission. Such evidence is essential to identify and assess potentials for improvement regarding effectiveness and efficiency of prehospital emergency care in Germany. One specialty of the German health care system is a high deployment of physicians in preclinical emergency medicine and a low transfer of authority to paramedics.

The objective of this study was therefore to provide a comprehensive description and comparison of hospital cases with and without the use of prehospital EMS based on a broad dataset of BARMER health insurance, which covers approximately 10.3% of the total German population.

## Materials and methods

### Data

We used health insurance data from the statutory health insurance provider BARMER, covering about 8.7 million individuals (approximately 10.3% of the German population) from all over Germany in 2022. We considered all hospital admissions of individuals insured with BARMER in 2022. For each hospital case, we retrieved information on age at admission, sex, main diagnosis (coded according to International Classification of Diseases, 10th revision, German modification; ICD-10-GM), patient clinical complexity level (PCCL), length of stay in hospital (LoS), hospital mortality (coded by discharge reason “death”) and selected procedures coded according to *Operationen- und Prozedurenschlüssel* (OPS; German modification of International Classification of Procedures in Medicine, ICPM). These procedures included intensive care complex treatment (OPS: 8‑980) and resuscitation (OPS: 8‑77). Resuscitation was also captured by diagnosis of “cardiac arrest with successful resuscitation” (ICD-10-GM: I46.0). We used information on the number of ventilation hours to identify patients ventilated during their hospital stay. In addition, we considered whether an individual was recognized as nursing home resident at the time of hospital admission. Data on costs per case for use of EMS and hospital treatment were derived from the respective billing positions.

### Types of emergency medical services

We considered five different types of EMS (Table 1). The use of these EMS by individuals on the day of hospital admission was identified based on billing data positions according to the German unified federal index of ambulance services (*Bundeseinheitliches Positionsnummernverzeichnis für Krankentransportleistungen*) [2].

**Table 1:** Classification of German emergency medical services (EMS)

English translation	German term	Description
Patient transport service ambulance (PTS)	Krankentransportwagen (KTW)	Van usually used for non-emergency transportation of patients
Double crewed ambulance (DCA)	Rettungswagen (RTW)	Van for the care and transportation of emergency patients
Physician-staffed Ambulance (PSA)	Notarzteinsatzwagen (NAW)	DCA with an emergency physician and additional equipment on board
Physician-staffed rapid response unit (PSRRU)	Rendezvous-System	Combination of DCA and an emergency physician, who is transported in a separate vehicle (Notarzteinsatzfahrzeug)
Helicopter emergency medical services (HEMS)	Luftrettung, Primärtransport	Aircraft (usually a helicopter) used for transportation of emergency patients from the emergency scene to a hospital (excluding transport from one hospital to another hospital)

### Inclusion and exclusion criteria

The sample included one day inpatient cases (German system: *teilstationäre Fälle*) and fully inpatient cases (German system: *vollstationäre Fälle*) admitted in 2022. We excluded cases that were transferred from another hospital. Cases transferred from another hospital were identified by admission reason “transfer” in the hospital data and coding of the respective EMS billing positions. The latter included interhospital air transports (German system: *Sekundärtransport—Luft*).

### Combined severity indicator

To assess the severity of hospital cases, we constructed a combined severity indicator. This indicator classified the severity of a hospital case as low or moderate if

1. the patient received only one day inpatient treatment OR
2. LoS was 3 days or less AND PCCL was 2 or lower AND the patient was not ventilated AND there was no ICU complex treatment AND there was no resuscitation AND the patient was discharged alive.

The severity of all cases neither fulfilling condition 1 nor condition 2 was classified as high.

### Statistical analysis

Data were analyzed descriptively. For categorical variables, we calculated absolute and relative frequencies. We used a kernel density estimator to analyze the distribution of age at hospital admission. We described the distribution of costs for use of EMS and hospital treatment by median and interquartile range. All analyses were stratified by use of EMS and EMS type. Statistical analysis was conducted with R (version 3.6.3) [4].

### Ethics

Approval of this study by an ethics committee was not required due to the use of fully pseudonymized, secondary data. Data preparation and analysis were conducted in line with the Guidelines and Recommendations for Good Practice of Secondary Data Analysis [7]. The study adheres to all relevant legal regulations, including the General Data Protection Regulation (GDPR), and national and international guidelines, including the Declaration of Helsinki.

## Results

### Patient characteristics

Approximately 569,000 (28%) of the 2.025 million hospital cases included in the sample used EMS in the context of hospital admission (Table 2). Most of these cases were transported by a double crewed ambulance (DCA), which accounted for more than 15% of all hospital cases.

**Table 2:** Hospital case characteristics and severity indicators by use and type of emergency medical services (EMS)

EMS	Number of cases (in 1000)	Share of cases (%)	Nursing home residents (%)	Ventilated cases (%)	ICU complex treatment (%)	Resuscitation (%)	Hospital mortality (%)	Low or moderate severity (%)
W/o EMS	1.456	71.9	1.2	1.4	0.7	0.3	1.1	51.4
PSA	23	1.1	10.1	7.7	3.3	2.7	9.3	33.8
PTS	117	5.8	21.0	2.1	1.4	0.7	7.2	21.3
DCA	305	15.1	11.1	2.7	1.9	0.8	5.7	30.3
PSRRU	121	6.0	10.0	8.4	3.8	3.1	10.0	31.0
HEMS	3	0.1	3.2	18.9	3.9	5.3	10.8	30.9

*PTS* patient transport service ambulance, *DCA* double crewed ambulance, *PSA* physician-staffed ambulance, *PSRRU* physician-staffed rapid response unit, *HEMS* helicopter emergency medical services, *W/o* without, *ICU* intensive care unit

**Fig. 1:**
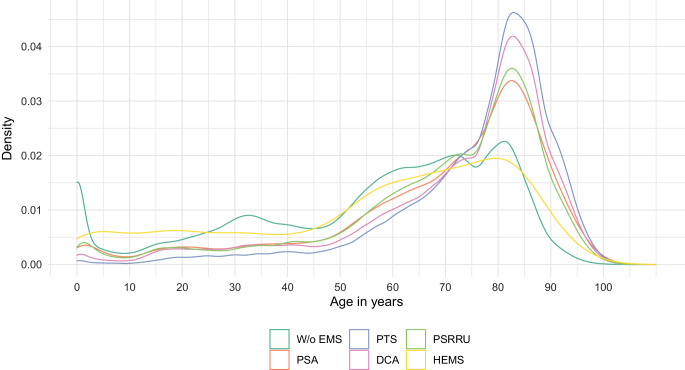
Age distribution of included cases by use and type of emergency medical services (EMS). The figure shows kernel density estimates. *PTS* patient transport service ambulance, *DCA* double crewed ambulance, *PSA* physician-staffed ambulance, *PSRRU* physician-staffed rapid response unit, *HEMS* helicopter emergency medical services, *W/o* without

The age distributions differed substantially between patients with and without use of EMS (Fig. 1). For all EMS except for helicopter emergency medical services (HEMS), there was a clear peak in the distributions between 80 and 90 years of age. In contrast, the distribution of patients transported with HEMS was more uniform across age groups and more similar to the age distribution of patients without use of EMS.

The proportion of nursing home residents was higher in EMS cases than in cases without use of EMS (Table 2). With more than 1/5 of all transported patients, patient transport service ambulance (PTS) showed the highest proportion of individuals living in nursing homes at the time of hospital admission. Nursing home residents accounted for approximately 1/10 of the patients with use of physician-staffed ambulance (PSA), DCA or physician-staffed rapid response unit (PSRRU).

### Clinical complexity and length of stay

**Fig. 2:**
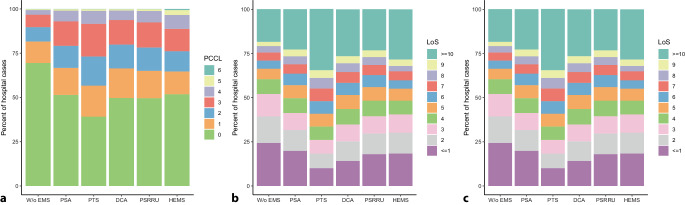
Distribution of patient clinical complexity level (PCCL) and length of stay in hospital (LoS) by use and type of emergency medical services (EMS). *PTS* patient transport service ambulance, *DCA* double crewed ambulance, *PSA* physician-staffed ambulance, *PSRRU* physician-staffed rapid response unit, *HEMS* helicopter emergency medical services, *W/o* without

Hospital cases with and without the use of EMS differed regarding the distribution of PCCL values and LoS (Fig. 2).

While the proportion of cases with a PCCL value of 0 was 69.5% when no EMS was used, it ranged between 39.2% (PTS) and 51.9% (HEMS) in cases with use of EMS (Fig. 2a). With 43.3%, the highest proportion of cases with severe clinical complexity (PCCL ≥ 2) was observed for PTS.

LoS was 3 days or less for more than half (52.1%) of the hospital cases without use of EMS (Fig. 2b). In cases with use of EMS, this proportion ranged between 26.1% (PTS) and 41.3% (PSA). The highest shares of cases with LoS of 10 days or more was observed for PTS (34.6%) and HEMS (28.4%). Excluding patients who deceased in hospital did not induce relevant changes in the distributions of LoS (Fig. 2c).

The proportion of cases with LoS of 1 day or less who did not decease in hospital was 24.2% in cases without use of EMS and ranged between 9.3% (PTS) and 16.9% (PSA) in cases with use of EMS.

### Severity

Generally, EMS cases showed a higher severity than hospital cases without use of EMS as measured by various indicators (Table 2). While 1.4% of all cases without use of EMS were ventilated in hospital, this was true for almost 19% of the hospital cases transported with HEMS. ICU complex treatment was also most frequently coded for cases transported with HEMS (3.9%), closely followed by PSRRU (3.8%). A similar picture emerged regarding resuscitation, which was most frequent in cases transported with HEMS (5.3%) and PSRRU (3.1%). For both of these EMS types, hospital mortality was 10% or higher. Cases transported with PSA ranked third with an in-hospital mortality rate of 9.3%. Interestingly, in-hospital mortality was lower in cases transported with PSA (5.7%) than in cases transported with PTS (7.2%).

According to the combined severity indicator, more than half (51.4%) of the cases without use of EMS in the context of hospital admission were characterized by low or moderate severity (Table 2). This proportion was lower for all types of EMS. However, more than 30% of the hospital cases with use of PSA, DCA, PSRRU, and HEMS did not exhibit one of the considered characteristics indicating high severity and, thus, were classified as “low or moderate”. The lowest proportion of cases with low or moderate severity was observed for PTS (21.3%).

### Most frequent main diagnoses by severity

The most frequent main diagnoses in hospital cases differed by severity and use and type of EMS (Tables 3 and 4).

**Table 3:** Ten most frequent main diagnoses (ICD 3) of hospital cases with **high severity** by use and type of emergency medical services

Rank	W/o EMS	PSA	PTS	DCA	PSRRU	HEMS
1	3.4%: M16 Coxarthrosis (arthrosis hip)	7.6%: I50 Heart failure	7.8%: I50 Heart failure	6.6%: S72 Fracture of the femur	9.1%: I50 Heart failure	9.7%: S06 Intracranial injury
2	3.3%: M17 Gonarthrosis (osteoarthritis of knee)	6.7%: I21 Acute myocardial infarction	3.6%: N39 Other disorders of the urinary system	6.1%: I50 Heart failure	7.7%: I21 Acute myocardial infarction	6.7%: I21 Acute myocardial infarction
3	2.8%: I50 Heart failure	6.3%: S72 Fracture of the femur	2.9%: S72 Fracture of the femur	5.4%: I63 Cerebral infarction	5.3%: J44 Other chronic obstructive pulmonary disease	6.3%: I63 Cerebral infarction
4	2.4%: F33 Recurrent depressive disorder	4.0%: J44 Other chronic obstructive pulmonary disease	2.6%: E86 Volume depletion	3.2%: N39 Other diseases of the urinary system	4.0%: S72 Fracture of the femur	4.4%: S82 Fracture of lower leg, including ankle
5	2.0%: F10 Mental and behavioral disorders due to the use of alcohol	3.8%: I63 Cerebral infarction	2.4%: J18 Pneumonia, organism unspecified	2.7%: E86 Volume depletion	3.5%: I63 Cerebral infarction	4.3%: S72 Fracture of the femur
6	1.4%: I70 Atherosclerosis	2.7%: J18 Pneumonia, organism unspecified	2.2%: S32 Fracture of the lumbar spine and pelvis	2.6%: J18 Pneumonia, organism unspecified	3.0%: J18 Pneumonia, organism unspecified	3.9%: S22 Fracture of rib(s), sternum and thoracic spine
7	1.4%: I63 Cerebral infarction	2.1%: I10 Essential (primary) hypertension	1.9%: J12 Viral pneumonia, not elsewhere classified	2.3%: J44 Other chronic obstructive pulmonary disease	2.5%: I48 Atrial fibrillation and flutter	3.2%: S32 Fracture of the lumbar spine and pelvis
8	1.4%: C34 Malignant neoplasm of bronchus and lung	2.0%: G40 Epilepsy	1.7%: J44 Other chronic obstructive pulmonary disease	2.0%: S32 Fracture of lumbar spine and pelvis	2.2%: G40 Epilepsy	3.0%: G40 Epilepsy
9	1.3%: F32 Depressive episode	1.9%: A41 Other sepsis	1.7%: N17 Acute renal failure	1.9%: J12 Viral pneumonia, not elsewhere classified	1.9%: A41 Other sepsis	2.5%: I50 Heart failure
10	1.3%: I48 Atrial fibrillation and flutter	1.9%: E86 Volume depletion	1.6%: I70 Atherosclerosis	1.7%: G45 Transient cerebral ischemic attacks and related syndromes	1.9%: J12 Viral pneumonia, not elsewhere classified	2.1%: S42 Fracture of the shoulder and upper arm

*PTS* patient transport service ambulance, *DCA* double crewed ambulance, *PSA* physician-staffed ambulance, *PSRRU* physician-staffed rapid response unit, *HEMS* helicopter emergency medical services, *W/o* without

**Table 4:** Ten most frequent main diagnoses (ICD 3) of hospital cases with **low or moderate severity** by use and type of emergency medical services

Rank	W/o EMS	PSA	PTS	DCA	PSRRU	HEMS
1	4.9%: Z38 Liveborn infants according to place of birth	6.2%: I10 Essential (primary) hypertension	4.8%: S06 Intracranial injury	8.9%: S06 Intracranial injury	6.4%: G40 Epilepsy	13.2%: S06 Intracranial injury
2	2.8%: I48 Atrial fibrillation and flutter	6.0%: G40 Epilepsy	3.0%: E86 Volume depletion	4.0%: I10 Essential (primary) hypertension	5.5%: I10 Essential (primary) hypertension	3.7%: I21 Acute myocardial infarction
3	1.9%: K40 Inguinal hernia	4.2%: S06 Intracranial injury	2.5%: S00 Superficial injury of head	3.6%: R55 Syncope and collapse	5.5%: I48 Atrial fibrillation and flutter	3.2%: S00 Superficial injury of head
4	1.8%: I25 Chronic ischemic heart disease	4.1%: I48 Atrial fibrillation and flutter	2.2%: I50 Heart failure	3.3%: S00 Superficial injury to head	5.0%: I21 Acute myocardial infarction	3.1%: I20 Angina pectoris
5	1.6%: K80 Cholelithiasis	3.9%: R55 Syncope and collapse	2.0%: N39 Other diseases of the urinary system	3.3%: I48 Atrial fibrillation and flutter	4.8%: I20 Angina pectoris	3.1%: S20 Superficial injury of thorax
6	1.3%: I20 Angina pectoris	3.4%: R07 Pain in throat and chest	1.9%: M54 Dorsalgia	2.9%: E86 Volume depletion	4.4%: R07 Pain in throat and chest	3.1%: S22 Fracture of rib(s), sternum and thoracic spine
7	1.3%: S52 Fracture of forearm	3.1%: I21 Acute myocardial infarction	1.9%: N13 Obstructive and reflux uropathy	2.9%: G45 Transient cerebral ischemic attacks and related syndromes	4.3%: S06 Intracranial injury	2.7%: G40 Epilepsy
8	1.2%: C50 Malignant neoplasm of breast	3.1%: I20 Angina pectoris	1.9%: I48 Atrial fibrillation and flutter	2.6%: F10 Mental and behavioral disorders due to the use of alcohol	3.7%: R55 Syncope and collapse	2.7%: I48 Atrial fibrillation and flutter
9	1.1%: F33 Recurrent depressive disorder	2.4%: F10 Mental and behavioral disorders due to the use of alcohol	1.8%: A09 Other gastroenteritis and colitis of infectious and unspecified origin	2.2%: H81 Disorders of vestibular function	2.3%: E86 Volume depletion	2.4%: S52 Fracture of forearm
10	1.1%: I70 Atherosclerosis	2.2%: E86 Volume depletion	1.7%: K56 Paralytic ileus and intestinal obstruction without hernia	2.1%: I63 Cerebral infarction	2.2%: I50 Heart failure	2.2%: S32 Fracture of the lumbar spine and pelvis

*PTS* patient transport service ambulance, *DCA* double crewed ambulance, *PSA* physician-staffed ambulance, *PSRRU* physician-staffed rapid response unit, *HEMS* helicopter emergency medical services, *W/o* without

In cases with high severity and without use of EMS, coxarthrosis and gonarthrosis ranked first and second, respectively, in the list of the most frequent diagnoses. In cases with use of EMS, heart failure, acute myocardial infarction, and fracture of the femur were represented in the top 10 diagnoses for most EMS types. In cases with high severity transported with HEMS, intracranial injury was the most frequent diagnosis.

Intracranial injury also ranked first in cases with low or moderate severity who were transported with HEMS, which reflects heterogeneity within the ICD-10-GM code S06 (Table 4). Other frequent main diagnoses in less severe cases with use of EMS were essential (primary) hypertension, epilepsy, volume depletion, and atrial flutter and atrial fibrillation.

### Costs

**Fig. 3:**
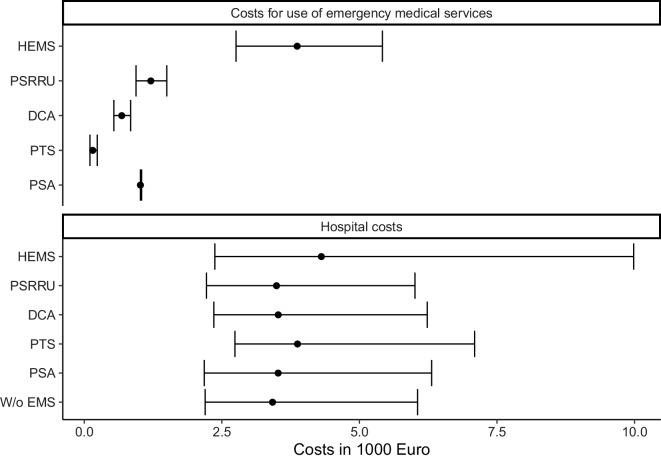
Median and interquartile range of costs per case for use and type of emergency medical services (EMS) and hospital treatment by use of EMS. *PTS* patient transport service ambulance, *DCA* double crewed ambulance, *PSA* physician-staffed ambulance, *PSRRU* physician-staffed rapid response unit, *HEMS* helicopter emergency medical services, *W/o* without

With a median of € 3900 (interquartile range [IQR] € 2800–5400), the costs per case for use of EMS were highest for HEMS (Fig. 3). The lowest median costs were observed for PTS (€ 150, IQR € 100–240). Median costs for hospital treatment were similar for cases without use of EMS and cases with use of PSA, DCA, or PSRRU. Relative to hospital cases without use of EMS, cases transported with PTS (+13%) and HEMS (+26%) had higher median costs of hospital treatment. As indicated by comparison of interquartile ranges, the heterogeneity in the distribution of costs was highest for HEMS regarding both use of EMS and hospital treatment.

## Discussion

This study presented relevant characteristics of cases with and without use of EMS in the context of hospital admission in Germany. Given the lack of previous national analysis and the current efforts to implement reforms of the German hospital and emergency care systems, such evidence is urgently needed for informed political decision making.

In detail, our results show that hospital cases with and without the use of EMS differed regarding multiple characteristics, including age distribution, clinical complexity, and various severity indicators. In this regard, the analysis also revealed relevant differences between EMS types. Taking multiple indicators into account, we found that the severity of more than 30% of the hospital cases with the use of EMS, except for PTS, was classified as “low or moderate”. More than 15% of all patients stayed one day or less in the hospital, and only half for more than 5 days. These findings may indicate that there is a relevant potential to avoid the use of EMS in the context of hospitalizations. This is of special interest, since the German health care system shows a high deployment of physicians in preclinical emergency medicine and a low transfer of authority to paramedics. Several main diagnoses such as atrial fibrillation, hypertension, a subgroup of heart failure patients and others (Tables 3 and 4) have substantial potential for outpatient treatment or treatment by general practitioner (GP) equivalents. This presumably demonstrates the incentives of the German DRG system to treat many patients in-hospital and may also reflect a deficits and access barriers in outpatient treatment. What stands out most, however, is the advanced age of hospitalized patients with use of EMS and the apparent lack of outpatient structures for elderly and very elderly patients. Given the high pressure for reform, the explorative evidence provided by this study should be extended and complemented by future studies focusing on potentials to improve the effectiveness and efficiency of the German EMS and health care systems.

Generally, our results are in line with previous analyses highlighting specific aspects of emergency care in Germany [8]. Regarding potentials to avoid the use of EMS in the context of hospital admissions, similar conclusions were drawn in related studies, e.g., on hospitalization of patients with seizures [1] or the use of EMS by elderly people for nonmedical reasons [6]. A study investigating predictors of hospital admission in patients using EMS in Munich, Germany found higher age to be one of the most important predictors of hospital admission [3]. Younger individuals were more likely to receive outpatient treatment. This result is consistent with our finding that individuals using EMS in the context of hospital admission were considerably older than hospital cases without use of EMS, with a peak in the age distribution between 80 and 90 years.

### Strengths and limitations

The main strength of this analysis is the use of health insurance data on more than 2 million hospital cases in 2022 from all over Germany. These data included information on use of EMS, diagnoses, procedures, costs, and multiple severity indicators such as ventilation or hospital mortality. This broad data allowed for comprehensive analysis and comparison of cases with and without the use of EMS regarding multiple patient and case characteristics.

A limitation of the data used in this analysis is that they do not include information on some clinical parameters (e.g., laboratory parameters or medical imaging) that may be relevant for assessing the severity of specific hospital cases. This may have induced overestimation of the proportion of cases with low or moderate severity. However, our combined severity indicator captures multiple relevant characteristics of severity based on diagnoses, procedures, and discharge reason. Hence, this indicator covers a broad range of relevant severity markers coded in health insurance data.

Furthermore, the data do not provide information on social or technical reasons that may justify the use of EMS in the context of hospital admission. Accordingly, the proportion of EMS cases with low or moderate severity should be interpreted with caution. For such cases, the use of EMS may have been necessary, although they did not show one of the characteristics captured by the combined severity indicator. Nonetheless, the high proportion of EMS cases with low or moderate severity indicates a relevant potential to reduce the burden on hospitals and ambulance services regarding patients seeking emergency care.

## Conclusions and suggested solutions

The current data suggest a substantial need of reforms of the German health care system addressing the lack of differentiation in the use of EMS, the guidance of patients in emergency care, ambulant care, the care of very old people in particular and the high conversion rate of patients admitted to the emergency department as in-patients. We therefore suggest implementing the following ideas, among others, to the upcoming reforms of the German healthcare system:

- Establishment of integrated control centers that ensure adequate allocation of patients seeking emergency care to care structures, e.g., emergency medical services (EMS) or ambulatory emergency service (German: *kassenärztlicher Notdienst*).
- Strengthening of primary care in the context of care for very elderly and care-dependent people (e.g., by community health nursing).
- If many emergency physicians continue to be deployed, they should also be able to provide treatment on-site without hospital admission in the future, as shown to be effective during the COVID 19 pandemic.
- To reduce the strong incentive of the German DRG system to admit patients as in-patients from the emergency departments.
- To formulate advanced care planning as a national goal and to adopt a patient-centered perspective on treatment and overtreatment.
- To explore the potentials of artificial intelligence in the preclinical emergency setting.

## Declarations

For this article no studies with human participants or animals were performed by any of the authors. All studies mentioned were in accordance with the ethical standards indicated in each case.
